# Sex-specific competition differently regulates the response of the rhizosphere fungal community of *Hippophae rhamnoides–*A dioecious plant, under Mn stress

**DOI:** 10.3389/fmicb.2023.1102904

**Published:** 2023-01-19

**Authors:** Yuhu Lin, Ling Fang, Hao Chen, Xudong Sun, Yunxiao He, Baoli Duan, Rui Li, Chuntao Cao, Juan Chen

**Affiliations:** ^1^Engineering Research Center of Chuanxibei RHS Construction at Mianyang Teachers’College of Sichuan Province, Mianyang Teachers’ College, Mianyang, China; ^2^School of Resources and Environmental Engineering, Mianyang Teachers’ College, Mianyang, China; ^3^Key Laboratory of Mountain Surface Processes and Ecological Regulation, Institute of Mountain Hazards and Environment, Chinese Academy of Sciences, Chengdu, China; ^4^Northwest Sichuan Geological Team, Sichuan Provincial Bureau of Geology and Mineral Resources Exploration and Development, Mianyang, China

**Keywords:** dioecious plants, sexual competition, Mn stress, fungal community, rhizosphere

## Abstract

In this study, we investigated the soil physicochemical parameters and responses of rhizospheric fungal communities of *Hippophae rhamnoides* to Mn stress under different sexual competition patterns. The results showed that competition significantly affects soil physicochemical properties, enzyme activity, and rhizosphere-associated fungal community structures. Under Mn stress, soils with intersexual competition had higher levels of N supply than those with the intrasexual competition. Moreover, fungal communities under intersexual interaction were more positive to Mn stress than intrasexual interaction. Under intrasexual competition, female plants had higher total phosphorus content, neutral phosphatase activity, and relative abundance of symbiotic fungi in soils to obtain phosphorus nutrients to alleviate Mn stress. In contrast, male plants had relatively stable fungal communities in soils. In the intersexual competition, rhizosphere fungal diversity and relative abundance of saprophytic fungi in male plants were significantly higher than in female plants under Mn stress. In addition, female plants showed greater plasticity in the response of rhizosphere microorganisms to their neighbors of different sexes. The microbial composition in soils of female plants varied more than male plants between intrasexual and intersexual competition. These results indicated that sex-specific competition and neighbor effects regulate the microbial community structure and function of dioecious plants under heavy metal stress, which might affect nutrient cycling and phytoremediation potential in heavy metal-contaminated soils.

## 1. Introduction

Industrialization and urbanization have led to ecological degradation and dramatic increases in heavy metals at regional and global scales due to anthropogenic and environmental factors (Zhong et al., 2016). Manganese (Mn) is a kind of heavy metal most widely used in industry and is also an essential trace element for plant growth and reproduction (Yao et al., 2012). Adequate amounts of Mn play an essential role in plant photosynthesis, activation of enzyme-catalyzed reactions, and maintenance of healthy cell organelle structure (Rayen et al., 2010; Bashir et al., 2013). The concentration of Mn required for normal plant growth and development is 20–40 mg/kg dry weight (Rayen et al., 2010). Mn toxicity symptoms usually appear when plants accumulate Mn above 150 mg/kg dry weight such as reduced photosynthetic efficiency, oxidative stress, damage to cellular ultrastructure, interference with the uptake of other nutrients, reduced biomass, and even death (Miyata et al., 2007; Rayen et al., 2010, 2012; Xie et al., 2015; Santos et al., 2017). Mn not only affects plant growth and development but also leads to further deterioration of environmental quality and even endangers human health by the food chain (Li C. et al., 2019).

Dioecious plant is an essential component of terrestrial ecosystems and plays an important role in maintaining species diversity and ecological stability (Hultine et al., 2016). The latest statistics show about 15,600 species of dioecious plants worldwide, belonging to about 175 families and 987 genera (Renner, 2014). Many morphological, physiological, and ecological differences have been observed in dioecious plants under environmental stresses, including drought, temperature, light, salinity, nutrient deficiencies, and heavy metal stresses (Chen M. et al., 2016, 2021; Liu et al., 2020, 2022a,c). Sexual differences in reproductive investment in plants may lead to sexual dimorphism in dioecious plants. Female plants in dioecious plants usually invest more in reproduction and less in growth than male plants (Juvany and Munné-Bosch, 2015; Li L. et al., 2019). Some literature reported that male plants of dioecious plants may grow better and exhibit higher tolerance in stressful environments than female plants due to differences in reproductive investment and ecophysiological responses (Lei et al., 2017; Melnikova et al., 2017; Liu et al., 2021a). The female plants of *Populus yunnanensis* displayed higher levels of ROS and weaker effective protection against excess zinc (Zn) conditions as compared to that of male plants (Jiang et al., 2013). Under Mn stress, male *Populus cathayana* plants were more resistant and tolerant than female plants (Chen et al., 2013). Liu et al. (2020) showed that female *P*. *cathayana* plants exhibited higher Cd uptake and root crown translocation capacity, while male plants showed more robust antioxidant capacity. Xia et al. (2020) showed that male *P*. *cathayana* had higher tolerance than female plants under low phosphorus nutrient stress by enhancing symbiosis with mycorrhizal fungi. Under the salt stress, the addition of ammonium and nitrate nitrogen promoted male *P*. *cathayana* efflux Na^+^ ions through the roots, and fewer Na^+^ ions were transferred to the shoot, which makes male plants have a higher salt tolerance than female plants (Liu et al., 2022b).

Competition and facilitation profoundly affect plant growth and environmental adaptation (Liancourt et al., 2005). Similarly, sexual competition patterns also significantly affected the stress tolerance of male and female plants under adversity. *Populus deltoides* females in intersexual competition grew faster than male plants, while males showed higher osmoregulatory capacity and antioxidant activity under salt stress (Li et al., 2016). Under herbivore stress, female *P*. *cathayana* in intersexual competition have better herbivore resistance than male plants, accumulating more secondary metabolites through the leaves (He et al., 2021). Under the intersexual competition between male and female plants, the high N growth stimulating effect was more significant for female *P*. *cathayana*; in contrast, male plants adapted more to intrasexual competition under low N (Chen et al., 2015). Compared with the intersexual competition, female plants in intrasexual competition accumulated more Cd and exhibited more injury under Cd stress (Chen J. et al., 2016). Pb stress and intrasexual competition had a more significant negative impact on growth and physiological parameters in female plants than in male plants (Chen et al., 2017). Most of the previous studies of dioecious plants have focused on the aboveground physiological response under stress, whether single-sex or sexual competition studies, while the response of rhizosphere microbial communities has been greatly neglected, especially under heavy metal stress.

*Hippophae rhamnoides* L. is a fast-growing dioecious woody plant widely distributed in northwestern China, characterized by drought tolerance and resistance to wind and sand, which plays a vital role in maintaining ecological stability. Studies have shown that *H*. *rhamnoides* can be used as an anti-pollution tree species for revegetation in heavy metal-contaminated mining areas (Yakun et al., 2016). However, the sex-specific response of *H*. *rhamnoides* to heavy metal stress in sexual competition patterns has mainly been neglected, especially regarding rhizospheric microbial community structure. Our study aims to address the following two questions: 1. Do sexual competition patterns and Mn stress affect rhizospheric soil physicochemical properties and enzymatic activities of *H*. *rhamnoides*? 2. How do sexual competition patterns and Mn stress affect rhizospheric fungal community composition and diversity?

## 2. Materials and methods

### 2.1. Plant materials and experimental design

Male and female seedlings of *H*. *rhamnoides* were collected from the Seedling cultivation base in Fuxin, Liaoning Province. The experiment was completely randomized with three factorial sex, Mn, and competition combinations. Two sexes (females and males), two Mn regimes (0, 4,000 mg Mn^2+^ kg^−1^ dry soil), and three competition treatments (female × female, FF; female × male, FM; male × male, MM) were used in the experiment. Corresponding intrasexual competition treatments are denoted as F/FF for females and M/MM for males, and intersexual competition treatments as F/FM for females and M/FM for males. The experiment was conducted in the Taiyi Xianshan Botanical Garden at Mianyang, Sichuan Province, China (31°27′ N, 104°49′E). On April 3, 2021, 160 healthy annual seedlings (80 females and 80 males) with relatively consistent growth were selected for planting. For interactions, two plants (two females, two males, or a female and a male) were cultivated 20 cm apart from each other in a plastic pot (external diameter 52 cm and height 35 cm) filled with 6.6 kg of homogenized soil of the same origin. Soil samples were air-dried and sieved through a 2 mm sieve. All pots were arranged randomly, and each treatment was replicated 10 times. After 8 weeks of growth, the plants were subjected to an Mn treatment for 12 weeks. The Mn treatments started on June 8, 2021, and the plants were harvested on September 11, 2021. In the Mn treatment, 100 mL of 486 mmol L^−1^ MnCl_2_·4H_2_O was evenly added to the pots every day during the first 10 days. The final Mn level reached 4,000 mg Mn^2+^ kg^−1^ dry soil, while the control plants were irrigated with equal quantities of deionized water (Chen J. et al., 2016). Throughout the experiment, the temperature range was 24–30°C during the daytime, 14–19°C during the nighttime, and 70–80% relative air humidity.

### 2.2. Soil sample collection and determination

The roots of *H*. *rhamnoides* were completely excavated, and the bulk soil of the roots was gently shaken off. Then the rhizosphere soil (soil closely attached to the surface of the root system about 2 mm) was collected in sterile bags with a sterile brush, frozen in liquid nitrogen and stored in an ultra-low temperature refrigerator at −80°C for rhizosphere soil microbial DNA extraction. The shaken off soil around the roots was also collected and divided into two parts, one stored at room temperature (25°C) for air-drying and the other stored at −20°C in the refrigerator for the determination of soil enzymatic activity.

Soil organic matter (SOM) content was determined by potassium dichromate oxidation - ferrous sulfate titration (Nelson and Sommers, 1996). Total nitrogen (TN) was determined by the Kjeldahl nitrogen method (Bremner, 1960). Ammonium (NH_4_^+^-N) and (NO_3_^−^-N) nitrate nitrogen contents were determined by 2 mol L^−1^ potassium chloride extraction-colorimetric method (Bao, 2000). Total phosphorus (TP) and available phosphorus (AP) content were determined by molybdenum blue colorimetric method (Olsen and Sommers, 1982). Determination of soil pH (the ratio of soil to water is 1:2.5) by an Acidimeter (PHS-3C; LEICI, Shanghai). Soil Mn content was determined by flame atomic absorption method. Soil enzyme activity was measured using the method described by Guan (1986). In brief, sucrase (SC) was determined by the 3,5 - dinitrosalicylic acid colorimetry method, and the sucrase activity was expressed as the mass (mg) of glucose released from 1 g of soil after 1 day; urease (UE) was determined by the phenol-sodium hypochlorite colorimetric method, and the urease activity was expressed as the mass (mg) of NH_3_^−^-N released from 1 g of soil after 1 day; neutral phosphatase (NP) was determined by disodium benzene phosphate colorimetric method, and the phosphatase activity was expressed as the mass (mg) of phenol released from 1 g of soil after 1 day; protease (PT) was determined by the ninhydrin colorimetric method, and the phosphatase activity was expressed as the mass (mg) of glycine released from 1 g of soil after 1 day. Soil enzymes were measured in fresh soil and converted to enzyme activity units per gram of dry soil by water content.

### 2.3. Fungal microbiome analysis

Soil microbial DNA was extracted from 0.5 g frozen soil samples using the Powerful Soil DNA Isolation Kit (MO BIO Laboratories, Carlsbad, CA, United States), following the kit instructions. The purity and integrity of DNA were detected by 1% agarose gel electrophoresis and Nanodrop (Nanodrop 2000, Thermo Fisher Scientific). The primers used for fungal ITS rRNA gene amplifications were ITS4 (5′- TCCTCCGCTTATTGATATGC-3′) and gITS7F (5’-GTGARTCATCGARTCTTTG-3′; Ihrmark et al., 2012). The PCR amplification reaction system and conditions were based on the previous study (Li et al., 2014). After PCR reactions, quality control, and purification processes, a library was constructed. All PCR products were sequenced on the Illumina NovaSeq platform (Illumina Inc., CA, United States) by Chengdu Institute of Biology, CAS. The raw data obtained by sequencing were spliced, quality filtered, and chimeras were removed to obtain effective data (Caporaso et al., 2010; Edgar et al., 2011). Based on the 97% sequence similarity level, all effective data were assigned to Operational Taxonomic Units (OTUs) using UPARSE pipeline (Edgar, 2013). Classification of fungal taxa was done using UNITE version 8.0 as a reference database (Kõljalg et al., 2005). FUNGuild functional annotation was used for predicting the ecological functions of fungal communities (Nguyen et al., 2016). The sequencing data were submitted to NCBI (BioProject accession number: PRJNA903369).

### 2.4. Statistical analyses

Statistical analysis was performed using the SPSS software package (version 25.0). Before ANOVAs, all data were checked for normality and the homogeneity of variances. Tukey’s test of one-way ANOVA analysis was used to determine the individual differences between the mean values. Independent-sample *t*-test was used to determine the significant differences between the control group and the Mn treatment group, and the significant differences were *P* < 0.05.

Based on the OTUs information, alpha diversity indices, including Observed species Chao1, richness, Shannon index, and Simpson index, were calculated with Qiime v1.9.0. Beta diversity was calculated based on Bray-Curtis distance metrics. Principal coordinates analysis (PCoA) was used to visualize the distribution of rhizosphere fungal communities in different treatment groups based on Bray-Curtis distance using the “Vegan” package in R. The PERMANOVA test was used to assess the percentage of variation explained by the Heavy metal, competition, sex, and their interactions on the rhizospheric fungal communities along with its statistical significance using the “Vegan” package. The linear discriminant analysis (LDA) effect size was performed to identify the significantly abundant taxa (phylum to genera) of fungi in different treatments, irrespective of sexual competition patterns (Class: Mn treatment; Subclass: sexual competition pattern), as well as in different sexual competition patterns irrespective of Mn treatment (Class: sexual competition patterns; Subclass: Mn treatment). Based on the FunGuild database, fungal functional groups were classified into symbiotroph, pathotroph, and saprotroph. Then, the relative abundance of the three functional groups was calculated, and their differences among the different treatment groups were analyzed by Tukey’s test and independent sample *t*-test. Pearson correlation analyses were performed to show relationships between soil environmental factors, fungal diversity, and fungal guilds. Prior to redundancy analysis (RDA), we used the ggvegan package to conduct a variance inflation factor (VIF) analysis for 12 soil environmental factors and then 10 soil environmental factors were used in RDA, TN, and Mn were omitted. The vegan and ggplot2 packages were used for RDA results to examine the correlation between soil environmental factors and fungal community changes.

## 3. Results

### 3.1. Soil physicochemical properties and enzyme activity

Under excess Mn conditions, the TP content of FF soil treatment was significantly 21 and 16% higher than MM and FM treatment, respectively, but there was no significant difference in the control group (Table 1). In addition, the TN content of FM soil treatment under excess Mn conditions was significantly 17% higher than FF, while in the control group, there was no significant difference between FM and FF, and the soil TN content of MM was the lowest. In control treatments, the soil in FF treatment showed 32 and 2% higher SOM content and pH than MM, respectively, while 20% lower the NH_4_^+^-N content than MM. Excess Mn treatment decreased the SOM content in soils from FF interactive pattern and NH_4_^+^-N content in the MM interactive pattern by 31 and 29%, respectively. In addition, the TN, AP, and Mn contents of all interaction patterns were significantly higher in the excess Mn treatment group than in the control group, and Mn content increased by 180, 213, 164%, in FF, MM, and FM, respectively (Table 1).

**Table 1:** Soil physical and chemical properties and enzyme activities under different competition and Mn treatments.

Soil properties	Control			Mn treatment		
	FF	MM	FM	FF + Mn	MM + Mn	FM + Mn
SOM (g kg^−1^)	32.59 ± 2.46a	24.71 ± 1.01b	24.78 ± 1.83b	22.43 ± 1.89A*	25.62 ± 1.64A	23.89 ± 0.78A
TN (g kg^−1^)	0.23 ± 0.01a	0.18 ± 0.01b	0.21 ± 0a	0.36 ± 0.02B**	0.34 ± 0.01B***	0.42 ± 0A***
TP (g kg^−1^)	0.54 ± 0.04a	0.51 ± 0.03a	0.49 ± 0.01a	0.57 ± 0.01A	0.47 ± 0.01B	0.49 ± 0.01B
NH_4_^+^-N (mg kg^−1^)	10.42 ± 0.33b	12.96 ± 0.44a	10.05 ± 0.24b	9.56 ± 0.43A	9.2 ± 0.61A**	10.71 ± 0.32A
NO_3_^−^-N (mg kg^−1^)	2.04 ± 0.1a	2.22 ± 0.24a	1.97 ± 0.17a	3.25 ± 0.42A*	2.09 ± 0.15A	2.54 ± 0.28A
AP (mg kg^−1^)	12.67 ± 0.95a	13.21 ± 0.32a	12.72 ± 0.49a	20.44 ± 1.18A**	17.23 ± 1.1A*	17.73 ± 0.38A***
pH	7.25 ± 0.04a	7.08 ± 0.02b	7.11 ± 0.02b	7.3 ± 0.03A	6.97 ± 0.04B*	7.01 ± 0.02B*
Mn (mg g^−1^)	0.45 ± 0.01a	0.46 ± 0.02a	0.45 ± 0a	1.26 ± 0.02AB***	1.44 ± 0.1A***	1.19 ± 0.03B***
SC (mg g^−1^ d^−1^)	24.67 ± 2.39b	35.81 ± 1.42a	24.04 ± 3.21b	46.54 ± 4.48A**	28.78 ± 2.06B*	38.55 ± 1.02AB**
UE (mg g^−1^ d^−1^)	1.45 ± 0.05a	1.25 ± 0.07a	1.21 ± 0.06a	1.27 ± 0.03B*	1.2 ± 0.03B	1.54 ± 0.05A**
NP (mg g^−1^ d^−1^)	0.37 ± 0.03a	0.45 ± 0.02a	0.47 ± 0.01a	0.42 ± 0.03A	0.24 ± 0.04B**	0.39 ± 0.01A**
PT (mg g^−1^ d^−1^)	0.59 ± 0.01ab	0.56 ± 0.01b	0.66 ± 0.03a	0.62 ± 0.02A	0.58 ± 0.02A	0.6 ± 0.01A

SOM, soil organic matter content; TN, total nitrogen content; TP, total phosphorus content; NH_4_^+^-N, ammonium nitrogen content; NO_3_^−^-N, nitrate nitrogen content; AP, available phosphorus content; Mn, manganese content; SC, sucrase activity; UE, urease activity; NP, neutral phosphatase activity; PT, proteinase activity. In the same row, different lowercase letters mean significant differences under control conditions, and different uppercase letters mean significant differences under Mn treatments according to Tukey’s test (*p* < 0.05). The asterisks indicate significant differences between control and Mn treatments within each competition treatment according to an independent-samples *t*-test (*p < 0.05, **p ≤ 0.01, ***p ≤ 0.001). Values are means ± SE (*n* = 4). FF soil from the female–female intrasexual competition; MM soil from the male–male intrasexual competition; FM soil from the female–male intersexual competition; FF + Mn soil from FF with Mn addition; MM + Mn soil from MM with Mn addition; FM + Mn soil from FM with Mn addition.

In Mn treatment group, the SC activity of FF was 62% higher than that of MM, while in the control group, it was 10% lower than that of MM (Table 1). In addition, the UE activity of FM was 21 and 28% higher than that of FF and MM under Mn treatment, respectively, and the NP activity of MM competition patterns was lowest under excess Mn conditions. However, both above enzymes in different interactive patterns were not significantly different in the control group. Moreover, UE activity increased by 27% under FM interactive pattern when excess Mn was used. In contrast, UE activity decreased by 12% in FF but remained stable in MM treatment. On the other hand, the PT activity of FM was significantly 18% higher than MM treatment in the control group; however, there was no significant difference in the Mn treatment group.

### 3.2. Composition of rhizosphere fungal community

No significant difference in the α-diversity of rhizospheric soil was observed in the control treatment (Table 2). The α-diversity of fungal communities was compared in rhizospheric soil from females and males under different competition patterns and excess Mn treatments (Table 2). There were no significant differences in Chao1 and Observed species in intra- and intersexual competition, except that in M/FM + Mn was significantly higher than M/MM + Mn treatment. In addition, the Shannon index and Simpson index were higher in M/FM + Mn than F/FM + Mn treatment, while there was no significant difference in intrasexual competition patterns. Compared with the control group, excessive Mn treatment significantly increased the Chao1 index of M/FM.

**Table 2:** Alpha diversity of fungi from the rhizospheric soil of *Hippophae rhamnoides* under different sexual competition patterns and Mn treatments.

	Competition pattern	Chao1	Observed species	Shannon index	Simpson index
Control	F/FF	1324.07 ± 82.3a	815 ± 36.69a	6.64 ± 0.06a	0.97 ± 0a
	M/MM	1338.52 ± 79.92a	820.75 ± 67.57a	6.61 ± 0.26a	0.97 ± 0.01a
	F/FM	1318.7 ± 56.82a	875.5 ± 26.01a	6.77 ± 0.19a	0.97 ± 0.01a
	M/FM	1311.85 ± 56.81a	895 ± 22.26a	6.92 ± 0.12a	0.97 ± 0.01a
Mn treatment	F/FF + Mn	1284.86 ± 60.44AB	827.75 ± 23.31AB	6.79 ± 0.09AB	0.97 ± 0A
	M/MM + Mn	1174.07 ± 123.89B	738 ± 69.12B	6.23 ± 0.32B	0.95 ± 0.01AB
	F/FM + Mn	1217.13 ± 48.06AB	773 ± 39.53AB	6.29 ± 0.14B	0.94 ± 0.01B
	M/FM + Mn	1506.78 ± 55.58A*	958.5 ± 30.88A	7.16 ± 0.11A	0.98 ± 0A

In the same column, different lowercase letters mean significant differences under control conditions, and different uppercase letters mean significant differences under Mn treatments according to Tukey’s test (*P* < 0.05). The asterisks indicate significant differences between control and Mn treatments within each competition treatment according to an independent-samples *t*-test (*p < 0.05, **P ≤ 0.01, ***P ≤ 0.001). Values are means ± SE (*n* = 4). F/FF indicates the rhizospheric soil from female plants in intrasexual competition; M/MM indicates rhizospheric soil from male plants in intrasexual competition; F/FM indicates the rhizospheric soil from female plants in intersexual competition; M/FM indicates the rhizospheric soil from male plants in intersexual competition. F/FF + Mn indicates the rhizospheric soil from F/FF under Mn treatment; M/MM + Mn indicates the rhizospheric soil from M/MM under Mn treatment; F/FM + Mn indicates the rhizospheric soil from F/FM under Mn treatment; M/FM + Mn indicates the rhizospheric soil from M/FM under Mn treatment.

**Figure 1:**
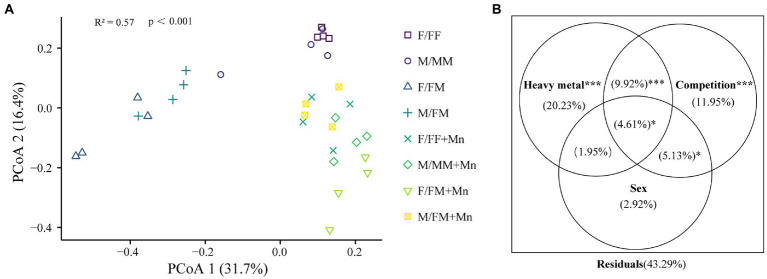
Principal coordinates analysis (PCoA) **(A)** and PERMANOVA results **(B)** based on Bray-Curtis distance metrics. Different colors or shapes represent different sample groups under different sexual competition patterns and Mn treatment; treatment codes are the same as in Table 2. Heavy metal: control, Mn treatment; Competition: intrasexual competition, intersexual competition; Sex: F, M. *0.01 < *p* ≤ 0.05; **0.001 < *p* ≤ 0.01; ***p ≤ 0.001.

As shown in Figure 1A, sexual competition patterns affected fungal community structure, and the composition of fungal communities was generally separated according to heavy metal and competition. Permutational multivariate analysis of variance (PERMANOVA) demonstrated that heavy metal was the largest source of variation (20.23%, *p* < 0.001; Figure 1B). The competitions were the second largest source of variation (11.95%, *p* < 0.001; Figure 1B).

**Figure 2:**
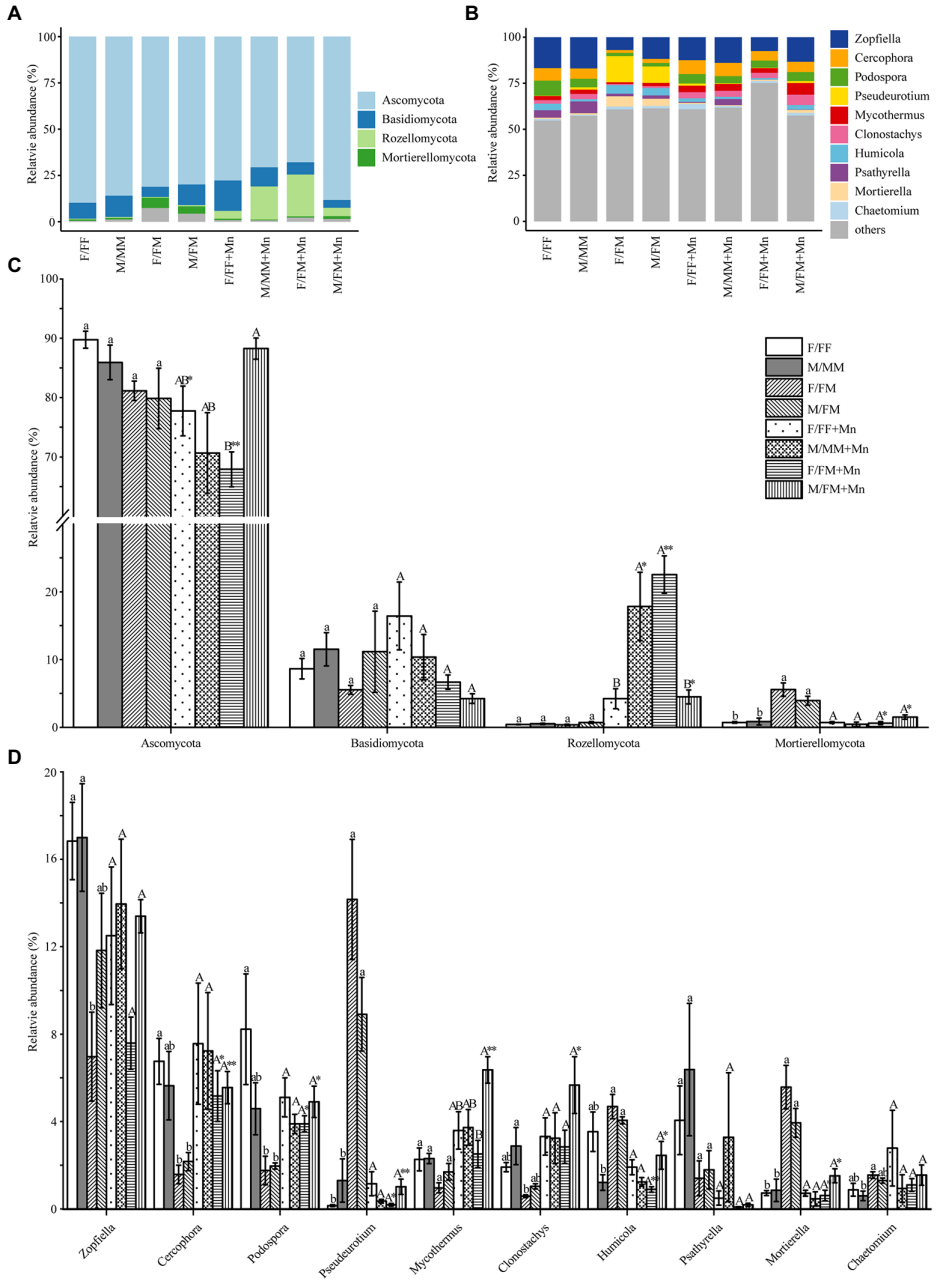
Relative abundances of dominant fungal phyla **(A**,**C)** and genera **(B**,**D)** under different sexual competition patterns and Mn treatment. Different lowercase letters mean significant differences under control conditions, and different uppercase letters mean significant differences under Mn treatments, according to Tukey’s test (*p* < 0.05). The asterisks indicate significant differences between control and Mn treatments within each competition treatment according to an independent-samples *t*-test (*p < 0.05, **p ≤ 0.01, ***p ≤ 0.001). Values are means ± SE (*n* = 4). Treatment codes are the same as in Table 2.

The taxonomic composition of fungal communities at the phylum level (relative abundance >1%) and genus level (top 10 relative abundance) is shown in Figures 2A,B. Ascomycota (68–90%) was an absolute dominant phylum in all treatments. Excess Mn reduced the Ascomycota phylum’s relative abundance in female plants’ rhizosphere in both inter- and intrasexual competition (Figure 2C). Basidiomycota, Rozellomycota, and Mortierellomycota were the dominant phyla with relative abundance greater than 1%. Excess Mn reduced the relative abundance of Mortierellomycota under intersexual competition. At the same time, the contrary was true for the Rozellomycota phylum, and females were significantly higher than males (Figure 2C). In addition, the relative abundance of Rozellomycota phylum was higher under excess Mn treatment than under control conditions in male form intrasexual competition. The abundances of the top 10 genera were in the following order: Zopfiella, Cercophora, Podospora, Pseudeurotium, Mycothermus, Clonostachys, Humicola, Psathyrella, Mortierella, Chaetomium (Figure 2B). Almost all fungal genera in intersexual competition differed between excess Mn treatment and control conditions, except Zopfiella, Psathyrella, and Chaetomium, while excess Mn treatment had no significant effect on all fungal genera in intrasexual competition (Figure 2D).

**Figure 3:**
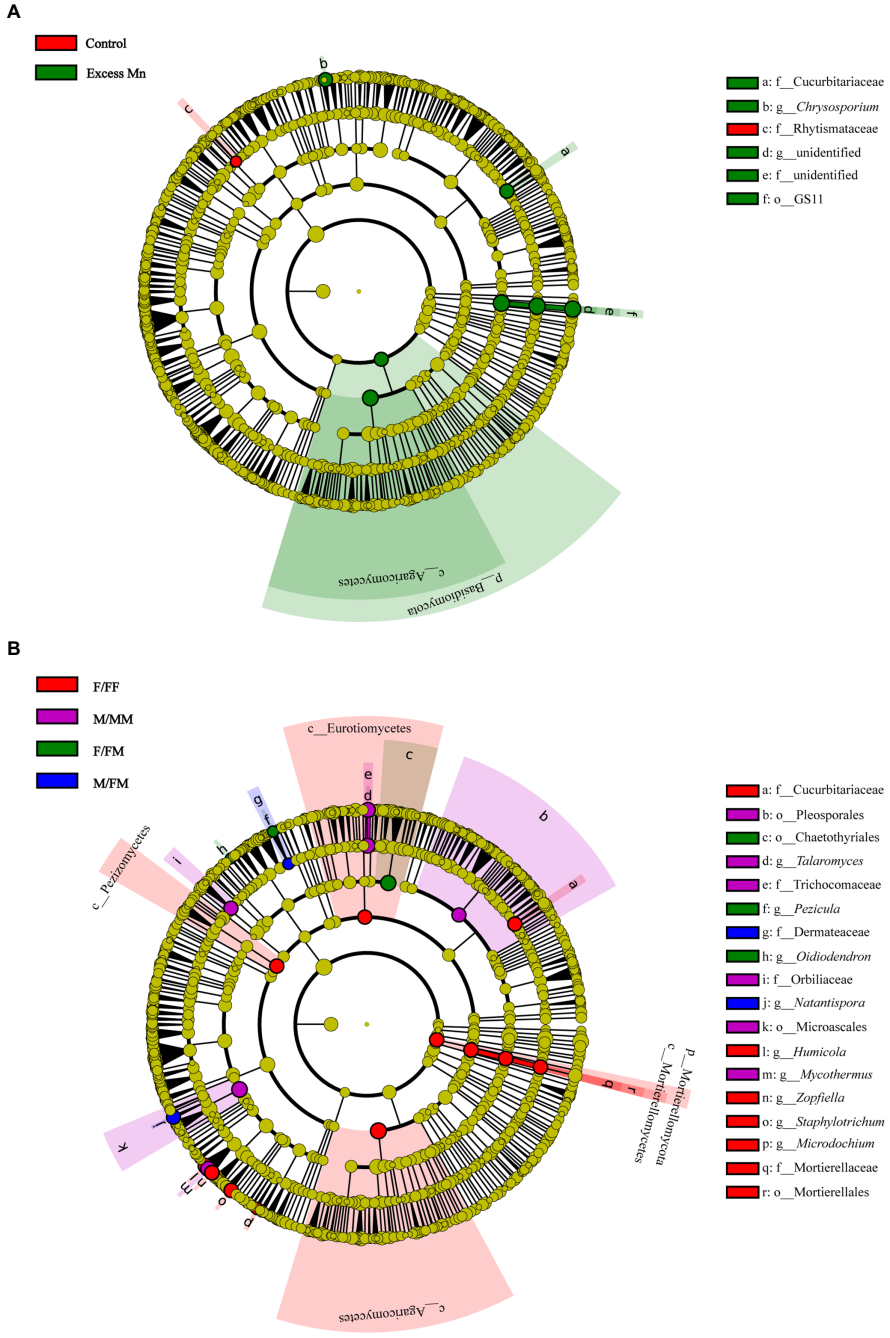
Fungal taxa with different abundance changes between control and Mn treatment, irrespective of sexual competition patterns **(A)**, and between sexual competition patterns, irrespective of Mn treatment as detected by the linear discriminant analysis effect size (LEfSe) analysis **(B)**. Only taxa with LDA over 5.5 are shown. The node color indicates taxa enriched under different treatment and interaction patterns.

The linear discriminant analysis (LDA) effect size analysis (LEfSe) was performed to compare the fungal composition from phyla to genera between Mn treatments, as well as between sexual competition patterns (Figure 3). We found that fungal compositions showed significant differences among sexual competition patterns and Mn treatments. The family Rhytismataceae was enriched in plants without excess Mn, while the phylum Basidiomycota, the class Agaricomycetes, the order GS11, the family Cucurbitariaceae, the genus *Chrysosporium* were predominant under excess Mn (Figure 3A). Irrespective of the Mn treatment, the phylum Mortierellomycota, the classes Mortierellomycetes, Agaricomycetes, Pezizomycetes and Eurotiomycetes, the order Mortierellales, the families Cucurbitariaceae and Mortierellaceae, the genera *Humicola*, *Zopfiella*, *Staphylotrichum* and *Microdochium* were abundant in the rhizosphere soil of F/FF, whereas the orders Pleosporales and Microascales, the families Trichocomaceae and Orbiliaceae, the genera *Talaromyces* and *Mycothermus* were more abundant in the rhizosphere soil of M/MM under intrasexual interaction (Figure 3B). In addition, the order Chaetothyriales, the genera *Pezicula* and *Oidiodendron* were dominant in F/FM under intersexual interactions, while the family Dermateaceae, the genus *Natantispora* were more abundant in M/FM (Figure 3B).

### 3.3. Fungal function guilds

**Figure 4:**
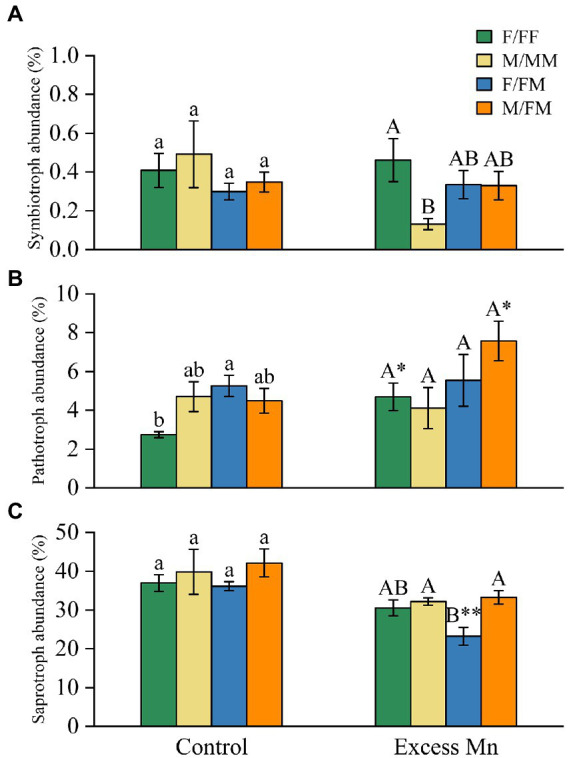
Relative abundances of symbiotrophs **(A)**, pathotrophs **(B)**, and saprotrophs **(C)** under different sexual competition patterns and Mn treatments. The asterisks indicate significant differences between control and Mn treatments within each competition treatment according to an independent-samples t-test (*P < 0.05, **0.001 < *P*  ≤ 0.01). Values are means ± SE (*n* = 4). Treatment codes and statistical significance codes are the same as in Table 2.

In the excess Mn treatment group, the relative abundance of symbiotroph in F/FF was significantly higher than M/MM, while there was no significant difference in intersexual competition (Figure 4A). The relative abundance of pathotroph in F/FF and M/FM was significantly higher in the excess Mn treatments group than in the control group (Figure 4B). In addition, excessive Mn treatment significantly reduced the relative abundance of saprotrophs in F/FM compared to the control and was significantly lower than M/MM and M/FM in the excess Mn treatment group (Figure 4C).

### 3.4. Associations between soil environmental factors and rhizosphere fungal communities

**Figure 5:**
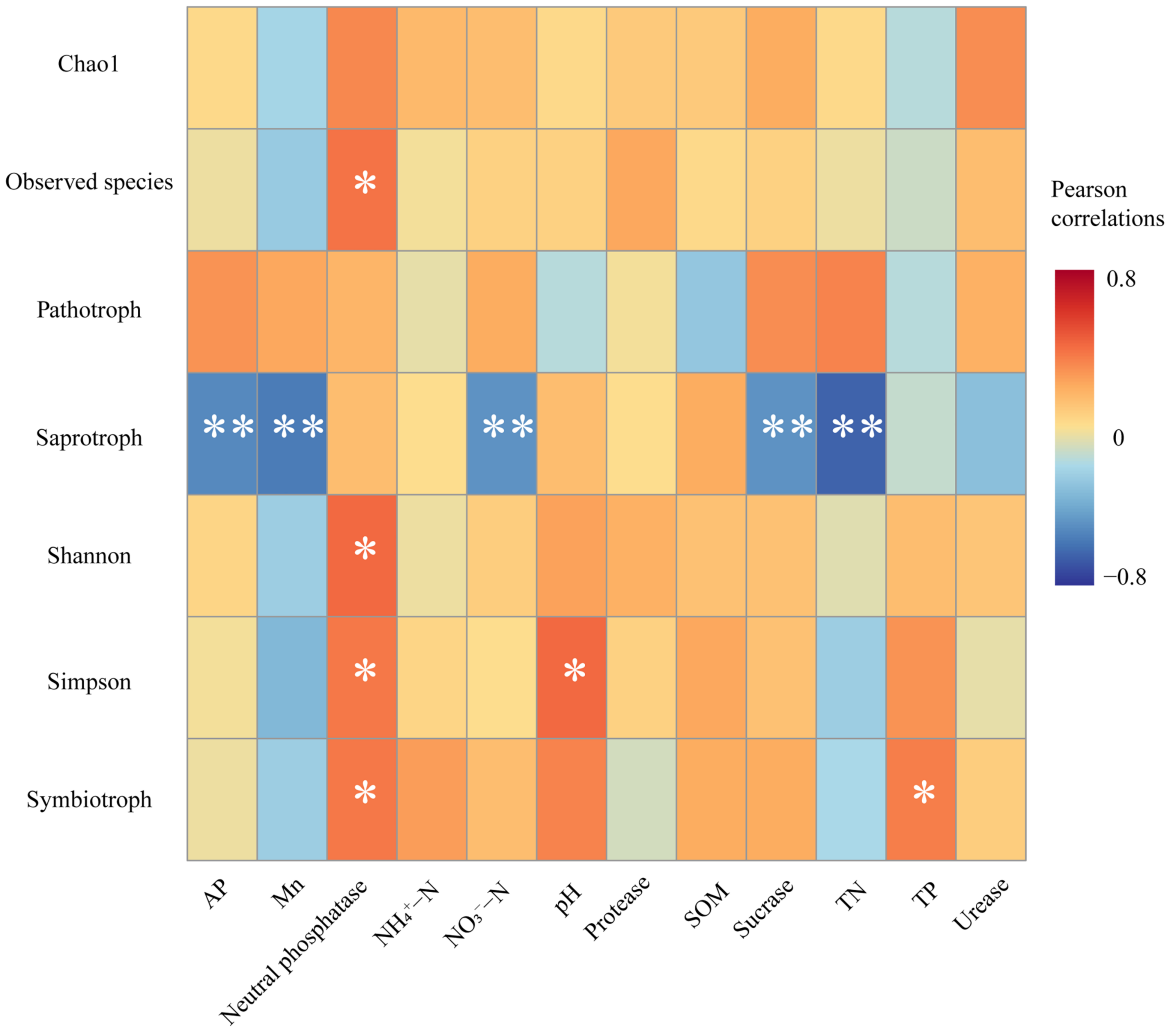
Pearson correlations between soil environmental factors, fungal diversity, and fungal functional guilds. Significances are marked as ***p ≤ 0.001; **p ≤ 0.01; *p ≤ 0.05.

The Pearson correlation heatmap showed that soil neutral phosphatase activities were significantly positively correlated with Observed species, Shannon index, Simpson index, and the relative abundance of symbiotrophs. The soil pH and TP content were significantly positively correlated with the Simpson index and symbiotrophs, respectively. In addition, the relative abundance of saprotroph was negatively correlated with many soil factors, including AP, Mn, NO_3_^−^-N, TN, and soil sucrase activities (Figure 5).

**Figure 6:**
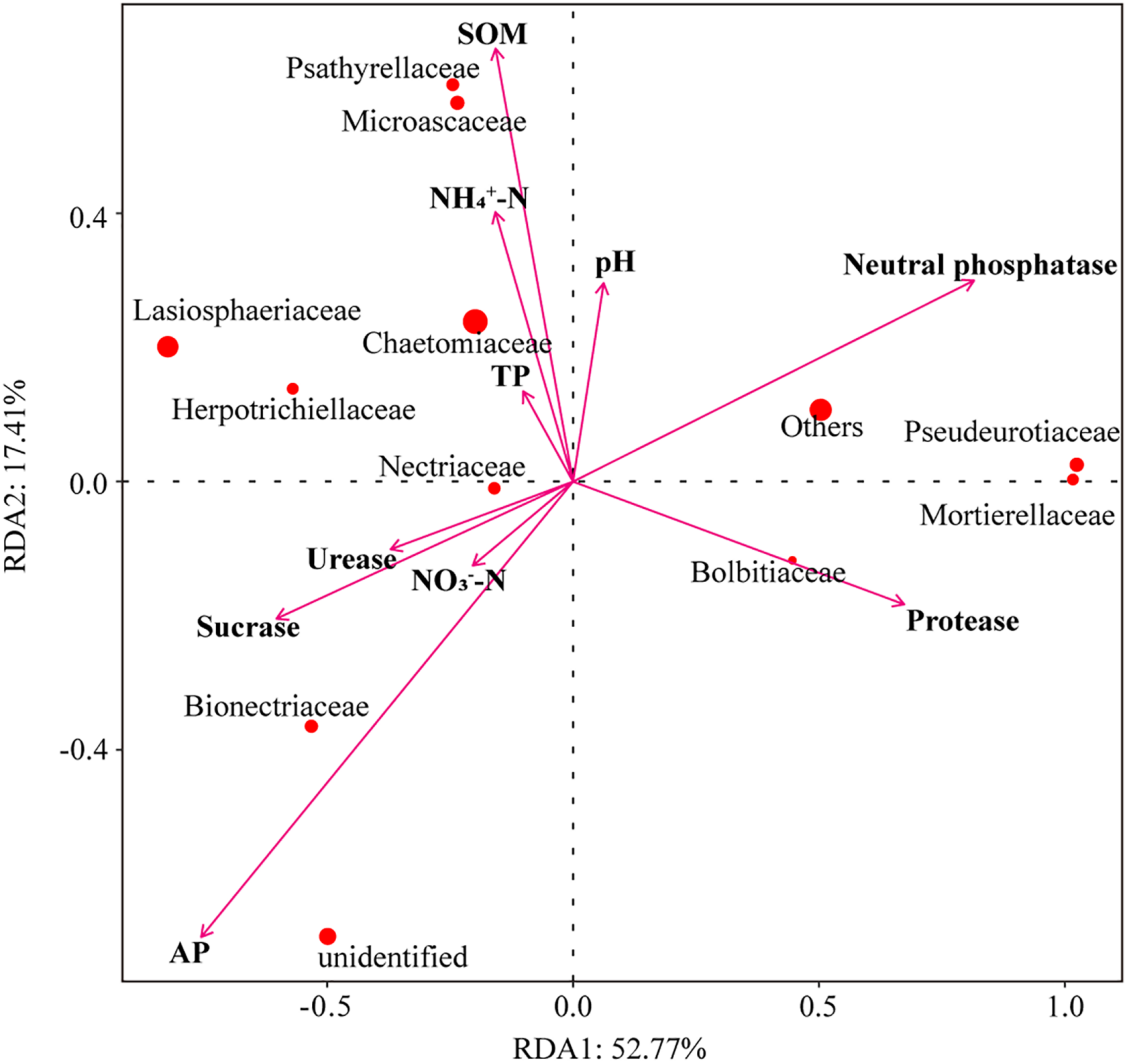
Redundancy analysis (RDA) of fungal families constrained by soil environmental factors across all experimental units. The arrows refer to soil environmental factors. The red circle represents fungal families, and the circle size represents relative abundance.

Collectively, the abiotic factors in Redundancy analysis (RDA) explained 70.18% of the variation in the relative abundances of fungal families. The first ordination axis RDA1 explained 52.77% and the second axis RDA2 explained 17.41% of the changes in the fungal communities (Figure 6). The results showed that SOM affected Psathyrellaceae, Microascaceaea, and Chaetomiaceae fungal communities. AP and sucrase affected Nectriaceae and Bionectriaceae fungal communities. Moreover, soil neutral phosphatase and Protease activities affected Pseudeurotiaceae, Mortierellaceae, and Bolbitiaceae fungal communities.

## 4. Discussion

### 4.1. Effects of sexual competition patterns and Mn stress on soil physicochemical properties and enzyme activities

Positive interspecific interactions among plants can improve soil conditions and promote plant nutrient uptake to enhance environmental tolerance (Shi et al., 2021). Previous studies have reported sex differences in nutrient element composition of male and female plant organs under different environments and competition patterns (Chen et al., 2014, 2017; Song et al., 2019). In this study, sexual competition patterns and Mn stress affected soil nutrients and enzyme activities. Under conditions of Mn stress and intrasexual competition, females had higher TP content, sucrase, and neutral phosphatase activity than males. It was also found that soil neutral phosphatase and TP content were significantly and positively correlated with Symbiotic fungi (Figure 5). When the supply of phosphorus in soil is insufficient, the symbiotic fungi dominated by mycorrhizal fungi accelerate the mineralization of organic phosphorus by secreting soil phosphatase to produce inorganic phosphorus for plants to absorb (Oehl et al., 2010). In addition, the AP content of the FF group was significantly higher by 61.3% after Mn addition compared to the control. The above results indicate that intra-female sexual competition effectively promotes the mineralization process of soil phosphorus, which can provide more phosphorus nutrients to plants. Interestingly, under Mn stress, the combined male and female treatments possessed the highest TN content and urease activity, indicating a higher level of nitrogen availability. It is possible that male and female plants in intersexual competition require more nitrogen nutrients to sustain plant growth. Plant competition can alter the availability and transformation of essential nutrients, which may affect plant responses to abiotic stresses and nutrient cycling in local ecosystems (Chen et al., 2017).

Previous studies have shown that soil enzymes play a vital role in soil ecological processes and can be used as effective indicators of the ecological impact of heavy metal contamination in soil (Šmejkalová et al., 2003; Paz-Ferreiro and Fu, 2013; Tang et al., 2019). In our study, females showed lower sucrase activity than males in intrasexual interaction under control treatments, while the opposite was true under Mn stress (Table 1). On the one hand, this may be related to the differences in resource utilization patterns between males and females (Juvany and Munné-Bosch, 2015). On the other hand, heavy metal stress may change the intensity of sex-specific competition (Chen et al., 2017).

### 4.2. Effects of sexual competition patterns and Mn stress on the structure and function of fungal communities

Soil microbes can spread horizontally through their surroundings, such as neighboring plants (Meyer et al., 2022). Rhizosphere and rhizoplane are important interfaces for microbial diffusion (Xiong et al., 2021). Heavy metals can also affect rhizosphere-driven microbial community structure (Hou et al., 2017). In addition, sex-specific interactions of dioecious plants have been shown to affect the composition of soil microorganisms (Xia et al., 2022). Our study further confirms this finding. We found that sexual competition patterns regulated the response of α-diversity of male and female *H*. *rhamnoides* rhizosphere fungi to Mn stress. In the control group, there were no significant differences in rhizosphere fungal diversity and richness in different sexual competition patterns, but significant differences were shown in Mn stress group. The fungal community richness of M/FM was significantly higher than that of M/MM under Mn stress. In addition, the diversity of the M/FM fungal community was significantly higher than that of F/FM under Mn stress. The root metabolites of M/FM under Mn stress may provide a broader niche for specific microbes adapted to particular substrates, and this puts its rhizosphere fungal community diversity and richness at a high level in the sexual competition (Xia et al., 2022). Soil physicochemical properties can affect fungal α- diversity (Berg and Smalla, 2009; Constancias et al., 2015). In this study, soil pH was significantly and positively correlated with the fungal Simpson index, which was similar to the studies of Yang et al. (2019) as well as Wang et al. (2020), and there were also studies showing that soil fungal community composition was highly significantly and positively correlated with soil pH (Shi et al., 2020). Elevated pH can reduce the availability of heavy metals and then mitigate damage to heavy metal-intolerant fungi, which may increase soil fungal diversity and change community structure. Compared to the control, Mn stress did not cause significant changes in rhizosphere fungal diversity and richness in male and female *H*. *rhamnoides*, except for Chao1 of M/FM. However, the fungal community structure was strongly affected by Mn treatment and competition and their interaction (Table 2), which probably involved root nutrients and Mn bioavailability (Liu et al., 2021b).

Vellend (2016) ecological community theory suggests that microbial community assembly is influenced by four evolutionary processes: dispersal, selection, diversification, and ecological drift. Selection effects due to the influence of biotic or abiotic factors can lead to changes in the abundance of microorganisms within the community (Cordovez et al., 2019; Fitzpatrick et al., 2020). In our study, Ascomycota was the absolute dominant phylum in all treatment groups. Ascomycota includes many saprophytic and parasitic fungi, mainly saprophytic fungi in soil, and can secrete a variety of cellulose and hemicellulolytic enzymes (Schoch et al., 2009; Baldrian et al., 2011). Mn stress significantly reduced the relative abundance of Ascomycota in female *H*. *rhamnoides* under intersexual and intrasexual competition (Figure 2C). At the same time, the relative abundance of saprophytic fungi was significantly lower in F/FM than in M/FM under Mn stress (Figure 4C). Therefore, we speculated that Mn stress inhibited carbon source metabolism of fungi in the rhizosphere soil of female *H*. *rhamnoides*. This inhibitory effect on females under intersexual competition was stronger than under intrasexual competition. In addition, the relative abundance of saprophytic fungi was higher in M/FM than in F/FM under Mn stress, which made M/FM more capable of organic matter decomposition and promoted above-ground plant growth. We also found that Agaricomycetes were enriched in the excess Mn treatments and F/FF groups (Figure 3). In contrast, most Agaricomycetes belonged to arbuscular mycorrhizal fungi and could provide essential nutrients and promote plant growth (Hibbett, 2006) Female *H*. *rhamnoide*s from the intrasexual competition will strengthen the association with symbiotic fungi such as Agaricomycetes to resist Mn stress by forming mycorrhizal structures in the roots, which can enhance P acquisition capacity to promote growth. Meanwhile, the relative abundance of F/FF rhizosphere Symbiotrops was significantly higher than M/MM under Mn stress (Figure 4A), which may make females from intrasexual competition more tolerant to Mn than males from the intrasexual competition by absorbing more phosphorus nutrition.

Previous studies have shown that neighboring plants have a greater impact on the performance of female plants, and the effect may be positive or negative (Graff et al., 2018). In our study, we found that F/FF and F/FM showed differential changes in the relative abundance of *Zopfiella*, *Cercophora*, *Podospora*, *Pseudeurotium*, and *Mortierella* under control conditions (Figure 2D). *Zopfiella* can control plant diseases by secreting antifungal compounds that inhibit the growth of plant pathogens (Huang et al., 2015), *Cercophora* can produce indole acetic acid and has the ability to dissolve and mineralize elemental phosphorus (Miranda et al., 2020), *Pseudeurotium* was a saprophytic fungus (Schadt and Rosling, 2015), the relative abundance of these three fungi in F/FF was higher than that in F/FM. However, the relative abundance of *Podospora* and *Mortierella* was significantly higher in F/FM than in F/FF. These two fungal taxa have important ecological functions in lignocellulose degradation and litter decomposition (Ellegaard-Jensen et al., 2013; Xie et al., 2014). Our results suggest that the rhizosphere microbes of *H*. *rhamnoides* females showed more plasticity in response to different sex neighbors. In contrast, the rhizosphere microbes of males are less sensitive to the sexual identity of neighbors. In addition, studies have shown that female plants from intra- and intersexual competition can distinguish the sexual identity of neighbors and change the investment models between growth and chemical defense (He et al., 2021; Zhang et al., 2021). We also found specific fungal colonization in the rhizosphere of *H*. *rhamnoides* under different sex competition patterns (Figure 3), which may result from the regulation of rhizosphere microorganisms by sexual competition patterns.

Furthermore, we found that male and female *H*. *rhamnoides* in the intersexual competition pattern were more positive to Mn stress than the intrasexual competition pattern in the rhizosphere microbial response (Figure 2D). Mn stress significantly increased the relative abundance of *Cercophora*, *Podospora*, *Mycothermus* and *Clonostachys* in the rhizosphere of intersexual competition of *H*. *rhamnoides*, which would enhance the cellulose degradation ability and disease resistance of *H*. *rhamnoides* (Che et al., 2002; Aquino et al., 2003; Cota et al., 2009). In addition, the relative abundance of *Pseudeurotium*, *Humicola* and *Mortierella* decreased, which may affect the accumulation of organic carbon and phosphorus mineralization (Ferrari et al., 2011; Clemmensen et al., 2015). However, *H*. *rhamnoides* in intrasexual competition was not affected by Mn stress. In the intersexual competition patterns, the mixed growth of male and female *H*. *rhamnoides* with different functions creates different rhizosphere microenvironments. Mixed sex planting increases microbial diversity in female and male rhizosphere (Xia et al., 2022). Previous studies have shown that resource complementarity and niche differentiation are fundamental mechanisms for improving ecosystem functioning (Loreau and Hector, 2001; Yu et al., 2022). We hypothesize that females and males in the intersexual competition patterns can create a more heterogeneous soil environment through resource complementarity or ecological niche differentiation to recruit more microbes at the rhizosphere than in intrasexual competition patterns. In addition, soil microbes are key drivers of changes in plant community structure and plant–plant interactions (Hodge and Fitter, 2013). This sex-specific adaptation and biochemical plasticity in dioecious plants are expected to lead to local adaptation and potential sex segregation (Charlesworth, 2002; Xia et al., 2022).

In summary, we believe that in the future production practice process, for male *H*. *rhamnoides*, intersexual competition is better than intrasexual competition; for female *H*. *rhamnoides*, intrasexual competition is better than intersexual competition.

## 5. Conclusion

The present study showed that Mn stress and sex competition strongly affected soil physicochemical properties, enzyme activity, and rhizosphere fungal abundance and diversity. Under Mn stress, there were significant differences in soil physicochemical properties and enzyme activities between male and female *H*. *rhamnoides* under different sex competition patterns, resulting in different nutrient availability. For example, F/FF in intrasexual competition patterns had a stronger soil phosphorus mineralization capacity and phosphorus supply level than M/MM. In contrast, intersexual competition FM soils had a higher nitrogen supply level under Mn stress. In addition, competition patterns and Mn treatment altered the structure of rhizosphere fungal communities of *H*. *rhamnoides*. In intersexual interaction, M/FM rhizosphere fungal diversity was significantly higher than F/FM under Mn stress. Females in intrasexual competition patterns can alleviate Mn stress by recruiting symbiotic fungi such as Agaricomycetes to obtain more P in symbiosis; in contrast, males in intrasexual competition patterns have a more stable microbial community to face Mn stress. In addition, females showed greater plasticity in the response of rhizosphere microorganisms to their neighbors of different sexes, and the rhizosphere microorganism of male and female *H*. *rhamnoides* to Mn Stress under intersexual interaction is more positive than intrasexual interaction. This study provides a new perspective for the remediation of heavy metal contaminated soil by *H*. *rhamnoides*, while more attention should be paid to the effect of sex interactions on dioecious plants in phytoremediation.

## Data availability statement

The datasets presented in this study can be found in online repositories. The names of the repository/repositories and accession number(s) can be found in the article/supplementary material.

## Author contributions

YL is responsible to experiment conduct, data collection and analysis, and manuscript writing. JC is responsible to complete experimental design and final manuscript. LF, XS, and HC are responsible to some data collect and analysis. YH, BD, RL, and CC are responsible to some data analysis. All authors have reviewed and approved the submission of our manuscript.

## Funding

This work was supported by the National Natural Science Foundation of China (No. 31500505), Sichuan Provincial Science and Technology Department Project (Nos. 2021YJ0293, 23NSFSC1968, 23NSFSC3100), and Postgraduate Innovation Fund Project (Nos. CX202204, CX202209).

## Conflict of interest

The authors declare that the research was conducted in the absence of any commercial or financial relationships that could be construed as a potential conflict of interest.

## Publisher’s note

All claims expressed in this article are solely those of the authors and do not necessarily represent those of their affiliated organizations, or those of the publisher, the editors and the reviewers. Any product that may be evaluated in this article, or claim that may be made by its manufacturer, is not guaranteed or endorsed by the publisher.
